# Cryopreservation of Mesenchymal Stem Cells Using Medical Grade Ice Nucleation Inducer

**DOI:** 10.3390/ijms21228579

**Published:** 2020-11-13

**Authors:** Nicholas M. Wragg, Dimitris Tampakis, Alexandra Stolzing

**Affiliations:** 1 Centre for Biological Engineering, School of Mechanical, Electrical and Manufacturing Engineering, Loughborough University, Loughborough LE11 3TU, UK; n.wragg1@keele.ac.uk (N.M.W.); dimitris.tampakis@kcl.ac.uk (D.T.); 2 Guy Hilton Research Centre, School of Pharmacy and Bioengineering, Keele University, Staffordshire, Stoke-on-Trent ST4 7QB, UK; 3 Regenerative Medicine, Division of Cancer Studies and Cancer Research UK King’s Health Partners, King’s College London, London SE5 9NU, UK; 4 SENS Research Foundation, Mountain View, CA 94041, USA

**Keywords:** mesenchymal stem cells, cryopreservation, ice seeding

## Abstract

Mesenchymal stem cells (MSCs) can differentiate into multiple different tissue lineages and have favourable immunogenic potential making them an attractive prospect for regenerative medicine. As an essential part of the manufacturing process, preservation of these cells whilst maintaining potential is of critical importance. An uncontrolled area of storage remains the rate of change of temperature during freezing and thawing. Controlled-rate freezers attempted to rectify this; however, the change of phase from liquid to solid introduces two extreme phenomena; a rapid rise and a rapid fall in temperature in addition to the intended cooling rate (normally −1 °C/min) as a part of the supercooling event in cryopreservation. Nucleation events are well known to initiate the freezing transition although their active use in the form of ice nucleation devices (IND) are in their infancy in cryopreservation. This study sought to better understand the effects of ice nucleation and its active instigation with the use of an IND in both a standard cryotube with MSCs in suspension and a high-throughput adhered MSC 96-well plate set-up. A potential threshold nucleation temperature for best recovery of dental pulp MSCs may occur around −10 °C and for larger volume cell storage, IND and fast thaw creates the most stable process. For adhered cells, an IND with a slow thaw enables greatest metabolic activity post-thaw. This demonstrates a necessity for a medical grade IND to be used in future regenerative medicine manufacturing with the parameters discussed in this study to create stable products for clinical cellular therapies.

## 1. Introduction

Mesenchymal stem cells (MSCs) are multipotent stromal cells that can differentiate into multiple different tissue lineages [1] making them an attractive prospect for regenerative medicine. However, a potentially more accessible application lies with their immune-modulatory activities and beneficial paracrine interactions [2]. MSCs can be isolated from many sources, including bone marrow and adipose tissue [3]. Dental pulp, from surplus extracted teeth, is a good source of highly proliferative MSCs without the technical limitations of other tissues. MSCs possess a multi-lineage differentiation capability and immunomodulation effect through production of molecules such as PGE2, IL-6 and nitric oxide. Through these mechanisms, MSCs have demonstrated possible positive effects against human age-related diseases [4] and have already reached the clinic for diseases like Alzheimer’s [5]. Minimal criteria to characterize MSCs were laid out by the International Society for Cellular Therapies (ISCT) in 2006 including, plastic adherence, tri-lineage differentiation (osteoblast, adipocyte, chondroblast) and positivity for surface markers of CD90, CD105 and CD73. CD 14, CD34 and CD45 must also be lacking expression in MSCs.

For commercial viability and patient access, it is necessary to have efficient, consistent, long term storage of MSCs. This will enable greater flexibility for storage and transport, and reduce costs of production through large batch manufacturing. Long term storage generally occurs at temperature less than −135 °C [6]. Yet, this cryopreservation can alter MSC function and consequently may lose their therapeutic potential [7,8]. There are several factors that interfere with cryopreservation, such as fluctuation in the temperature of ice nucleation, inadequate cryopreservation agent concentrations and thawing rate [9]. This can lead to cellular damage from osmotic imbalance, lethal intracellular ice formation, exposure to toxic concentrations of cryoprotective agents during freezing and oxidative stress during thawing (potentially as a result of osmotic imbalances) and after. These effects are felt within the first 36 h of culture following thaw. Further reading on cryopreservation stress factors and their resulting effects were discussed by Baust et al. (2009) [10]. It is therefore vital to improve the cryopreservation process to reduce variability and negative MSC population function effects.

As one of the greatest influencers in freezing rate instability, ice nucleation is characterized by the change in state of the cryosolution from liquid to solid [11]. As the temperature of the cell suspension reduces, latent heat in the system allows the solution to remain liquid below the freezing point of water. At a certain temperature defined by the roughness of the cryovessel surface and salt content and disturbances in the cryosolution, the phase change happens [12,13,14,15]. During this stochastic process, energy, in the form of heat, is released, causing a rapid rise in temperature back towards 0 °C, followed by a rapid reduction [13,16]. The lower the temperature of ice nucleation occurs, the greater the change in the rate of cooling. Ice seeding (the addition of components to induce ice nucleation) has demonstrated the potential to induce ice formation at temperatures greater than −10 °C which could reduce the chaotic effects during freezing and allow for a better controlled manufacturing process [13,17,18,19,20,21].

A less controlled area of cryopreservation is the thawing rate. It is widely assumed that a fast thawing rate (less than 5 min) reduces the chances of localized re-freezing causing damage to the cells [22]. Conventional procedure is to use a water bath in which user/protocol variability has a large influence on cell viability. Recently, in tandem with controlled rate freezers, controlled rate thawing has been applied to cryovials and cryobags when considering cells for use in therapies, although the accepted thawing rate is still high. While this has now been applied to testicular interstitial cells, pluripotent and mesenchymal stem cells [23,24] frozen in cryovials, there are no data on cryopreservation of MSC in a 96-well plate format. This could be used in developing cryosolutions or as a pre-prepared plate for toxicology testing or rapid distribution of cells. This manuscript seeks to enhance our understanding of the effects of using (1) a new ice seeding device, (2) plate freezing of adhered cells and (3) thawing rates, on cell viability, metabolic activity and differentiation capability as part of a wider manufacturing process.

## 2. Results

### 2.1. Ice Nucleation Temperature

**Figure 1:**
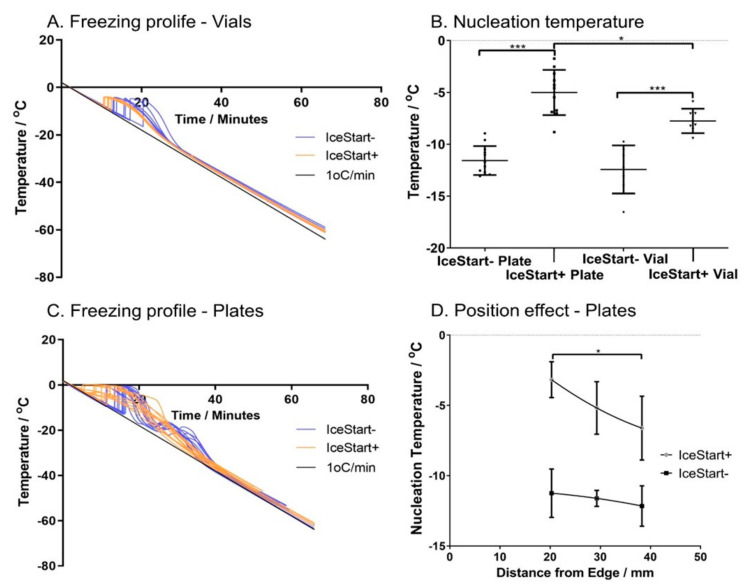
Freezing profiles and nucleation temperature. (**A**) Cryovial cryosolution temperature change over time with (orange) and without (blue) an ice nucleation device (IND). (**B**) Nucleation temperature of cryosolution in different conditions (IND+/-) in both 96-well plates and cryovials. (**C**) The 96-well plate cryosolution temperature variation over time IND+: Orange, IND-: Blue. (**D**) Cryosolution nucleation temperature variation due to well distance from edge IND+/-. (Vials: *n* = 4 replicates + 3 repeats per condition. 96-well plates: *n* = 4 repeats of three well positions. * *p* ≤ 0.05, *** *p* ≤ 0.001, bars represent ± SD).

Changes in the temperature of ice formation due to the inclusion of an ice nucleator were measured during controlled rate freezing at 1 °C/min. In cryovials with 1.0 mL of cryostorage solution, a characteristic spike in the solution temperature, associated with the change in phase, was observed over a range of approximately 7 °C from −9.7 °C to −16.5 °C. This resulted in a maximum cooling gradient of −2.64 ± 0.67 °C/min to return to the continuing 1 °C/min (Figure 1A). A slight deviation from the intended cooling rate in addition to the effects of the phase change is observed from inefficiencies in the sterling engine heat transfer as the differential in internal and ambient temperature increase. With the inclusion of an ice nucleator, the mean nucleation temperature is increased significantly to a range of −5.9 °C to −9.4 °C with a reduced standard deviation (Figure 1B). This also results in a reduced max cooling rate of −2.16 ± 0.05 °C/min with an accompanying 10-fold reduction in coefficient of variation over the IceStart− condition.

In 96-well plates, the cooling profiles demonstrate an initial spike in temperature before decreasing rapidly towards the 1 °C/min cooling rate, followed by a secondary thermal instability at approximately −20 °C (Figure 1C). Inclusion of the ice nucleation device (IND) into the wells significantly increased (*p* < 0.001) the ice formation temperature (Figure 1B) from −11.57 ± 1.39 °C to −5.01 ± 2.18 °C. Interestingly, the variation in the 96-well plate normal conditions (IceStart−) were lower than in vials without an IND and, in contrast to the cryovials, variation was increased in the 96-well plates with the addition of the IND. Cooling in the controlled-rate freezer is done by direct contact with a cold plate and conducting surface to the wells; in 96-well plates, no significant differences were observed in well position in IceStart− conditions (Figure 1D). With the addition of the IND, those wells closer to the edge of the plate had a significantly higher temperature of ice formation. This is possibly due to the overall increased mass with the addition of the device, and therefore an increase in the latent heat to be removed for each well closer to the centre of the plate. Despite an increased variation with an IND, the maximum cooling rate is significantly reduced (*p* < 0.001) from −5.73 ± 1.07 °C/min (IceStart−) to −2.57 ± 1.12 °C/min, although the range remains approximately consistent regardless of the mean shift (IceStart−: −7.6 to −4.2 °C/min, IceStart+: −5.4 to −1.6 °C/min). It is important to note that the nucleation temperature is significantly increased with the addition of the IND despite the presence of the thermocouples which act as an additional source of ice nucleation.

### 2.2. Effect of Thawing with Ice Nucleation Device

**Figure 2:**
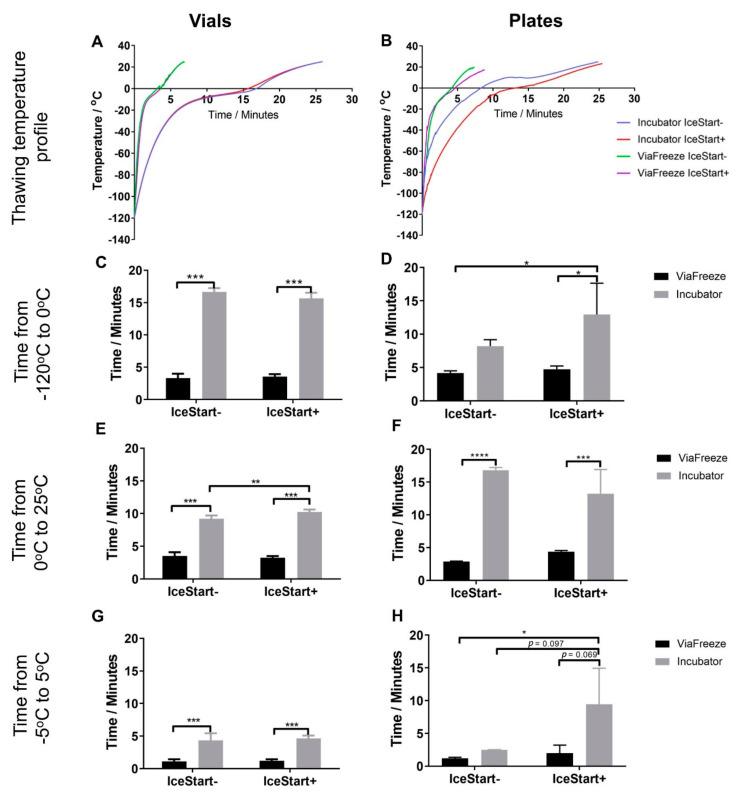
Thawing profiles and time across significant temperature zones. Vial (**A**) and 96-well plate (**B**) cryosolution temperature during warming (fast warming provided by ViaFreeze device, slow warming provided by incubator). Slow thaw/IND-: Blue, Slow thaw/IND+: Red, Fast thaw IND-: Green, Fast thaw IND+: purple (**C**,**D**) Time from long-term storage temperature to theoretical melting point. (**E**,**F**) Time from theoretical melting point to room temperature towards acceptable culture temperature. (**G**,**H**) Time across phase change temperature range—solid to liquid. (*n* = 2 replicates + 3 repeats for each condition * *p* ≤ 0.05, ** *p* ≤ 0.01, *** *p* ≤ 0.001, **** *p* ≤ 0.0001, bars represent ± SD). Legend applies to both graphs on row.

Thawing rates were also investigated to ascertain whether overall rate of temperature change, timing of phase change or time from 0 °C were affected by the IND (Figure 2). A cell culture incubator set to 37 °C was used as a slower method of thawing (warming from ambient temperature) and the heating function of the controlled rate freezer at 37 °C as a fast thawing method. Within the cryovials, the extra mass of the IND only altered the heating time significantly from 0–25 °C with ambient heating in the incubator. This difference, whilst statistically significant, can be considered negligible with only a 14.83 s difference. Contrastingly, the increased mass of the IND array in the 96-well plates caused a significant difference in time to 0 °C and a large difference in the means over the phase transition (−5 to 5 °C). The variability, potentially due to the well position, renders this difference to be not statistically significant (*p* = 0.097).

**Figure 3:**
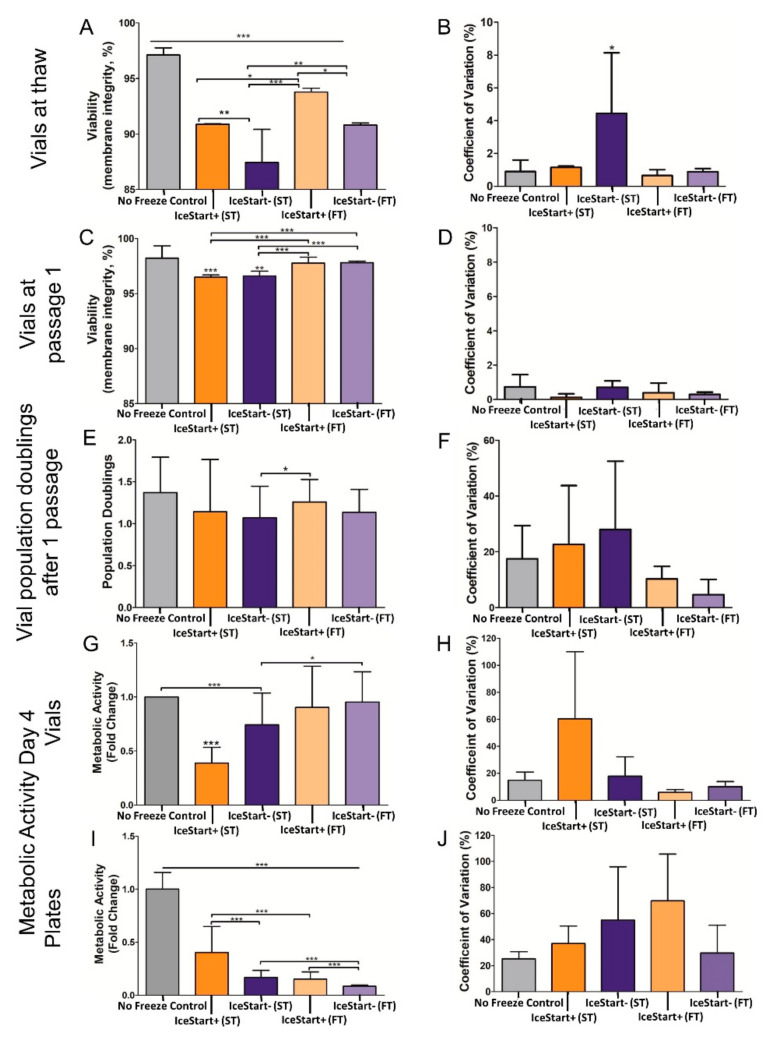
Mesenchymal stem cell (MSC) recovery characteristics using IND+/-, Slow Thaw (ST) and Fast Thaw (FT) conditions. (**A**) Viability measured through membrane integrity at thaw in vials. (**B**,**D**,**F**,**H**,**J**) corresponding coefficient of variation. (**C**) Viability at first passage following thaw in vials. (**E**) Population doublings after 1 passage from cryostorage in vials. Metabolic activity at four days (passage 1) following thaw in vials (**G**) and 96-well plates (**I**). (Vials—*n* = 6 replicates + 3 donor repeats for each condition. 96-well plates—*n* = 48 replicates + 3 donor repeats for each condition. * *p* ≤ 0.05, ** *p* ≤ 0.01, *** *p* ≤ 0.001, bars represent ± SD).

In line with this significant increase in nucleation temperature with an IND, viability, as measured by membrane integrity, was significantly greater (*p* ≤ 0.05) in IceStart+ conditions (Figure 3). The slow thaw (ST) conditions had a significantly reduced viability in comparison to the more standard fast thaw (FT) conditions; although, the IND demonstrates a similar result to the fast thaw IceStart− conditions. This suggests that the less variable cooling may help to ensure membrane integrity and assists in the controlled nature of the thaw. In terms of variability, ST IceStart− conditions demonstrated the greatest proportional variation (coefficient of variation) with no other significant differences occurring.

After one passage, although the fast thaw conditions were significantly greater (*p* ≤ 0.001), all conditions had a viability greater than 95%. A similar relationship is again observed in each donor and no significant differences in variation. However, in cell number, IceStart+ FT conditions had a greater mean yield although the variation across all other condition suggests that this is not significant. Between IceStart+ ST and IceStart− FT, cell numbers were not significantly different again suggesting that the IND may be causing greater variation in the cryopreservation process. However, slow thaw conditions appear to negatively affect the metabolic activity of the cell populations with IceStart+ ST conditions affected the most (Figure 3G). This may be a consequence of a slower thaw causing osmotic imbalances. Despite no significant differences, the metabolic activity associated co-efficient of variation was also lowest in the IceStart+ FT conditions.

In 96-well plate conditions, where the cells are attached prior to freezing, ST conditions had significantly greater metabolic activity. IceStart+ conditions demonstrated approximately double the metabolic activity of their IceStart− counterparts but IceStart+ ST was only approximately 40% of the no freeze controls. This substantial difference is most likely attributed to changes in the plate surface (size and mechanical properties) which caused cell detachment and potentially damage. Unlike in vial recovery conditions, the greatest co-efficient of variation associated with metabolic activity was in the IceStart+ FT conditions; however, this is most likely due to the very low recovery in the IceStart− FT condition.

**Figure 4:**
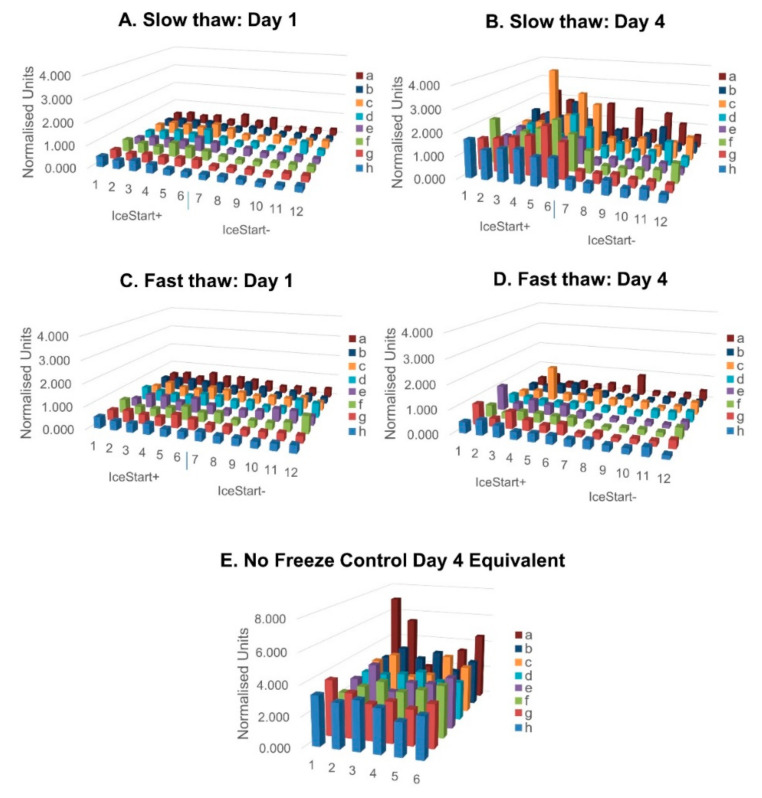
Metabolic activity normalised to Day 1 No Freeze control values for each well, respectively. IceStart+ conditions demonstrate greater metabolic activity following thaw with slow thaw conditions recovering to the greatest extent. *n* = 3 donor repeats. (**A**) Day 1 post thaw MTT slow thaw, (**B**) Day 4 slow thaw, (**C**) fast thaw Day 1, (**D**) Day 4 fast thaw, (**E**) Day 4 equivalent No Freeze control. *n* = 3 donor repeats for each condition.

**Figure 5:**
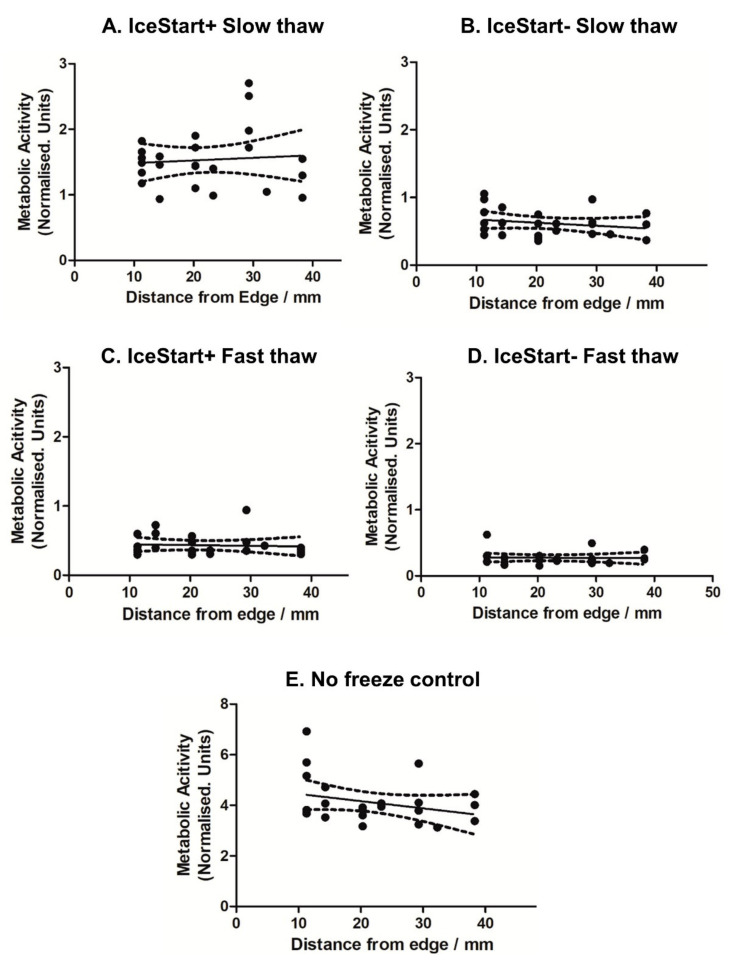
Linear regression with 95% confidence intervals of Day 4 metabolic activity (normalised to Day 1 No Freeze control values for each well respectively). (**A**) IceStart+ slow thaw (ST), (**B**) IceStart− ST, (**C**) IceStart+ fast thaw (FT), (**D**) IceStart− FT, (**E**) No Freeze control. Frequency of well position values vary due to different replicate positions across a 96-well plate. Mean values calculated from *n* = 3 donor repeats for each well position.

A total of 24 h after thawing in 96-well plates, similar metabolic activity was observed in both slow and fast thaw conditions (Figure 4) although in fast thaw conditions, greater activity is observed in the IceStart− (IS- FT) condition than in the IS- ST. No significant well position bias (Figure 5) is apparent although the no freeze control suggests a slight reduction in metabolic activity towards the centre of the plate (Figure 5E). This shows that despite a significant difference in well position and the temperature of ice nucleation across a plate (Figure 1D), this has no effect on the recovery of the cells, potentially indicating a threshold nucleation temperature for best recovery of dental pulp MSCs around −10 °C. A reduction in metabolic activity across the four days, in some wells of the FT IS- condition, indicates that damage is occurring as a result of the thawing. With the combination of IS+ and slow thaw demonstrating greatest recovery in continued culture, the IND may offer protection during the freezing process. This is possibly due to minimized possibility of temperature gradients in a small volume of medium. A slower thaw may then cause the expansion rate of the culture surface to happen over a longer time period (as a solid, warming at a different rate to the liquid), enabling the cell to adjust.

### 2.3. Differentiation Ability of MSC after Freezing with IceStart

**Figure 6:**
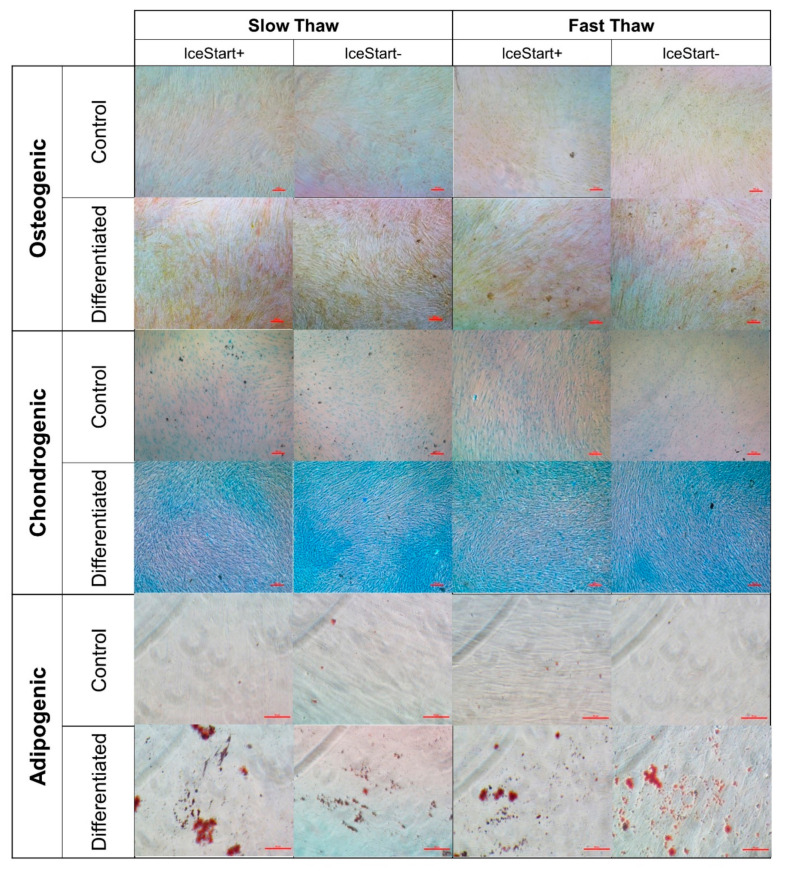
Representative images of MSC tri-lineage differentiation. No inhibition of differentiation was observed in any condition (3 donors). Control is stain control (without culture in differentiation medium). Osteogenic and chondrogenic conditions scale bar: 100 µm, Adipogenic conditions scale: 50 µm.

**Figure 7:**
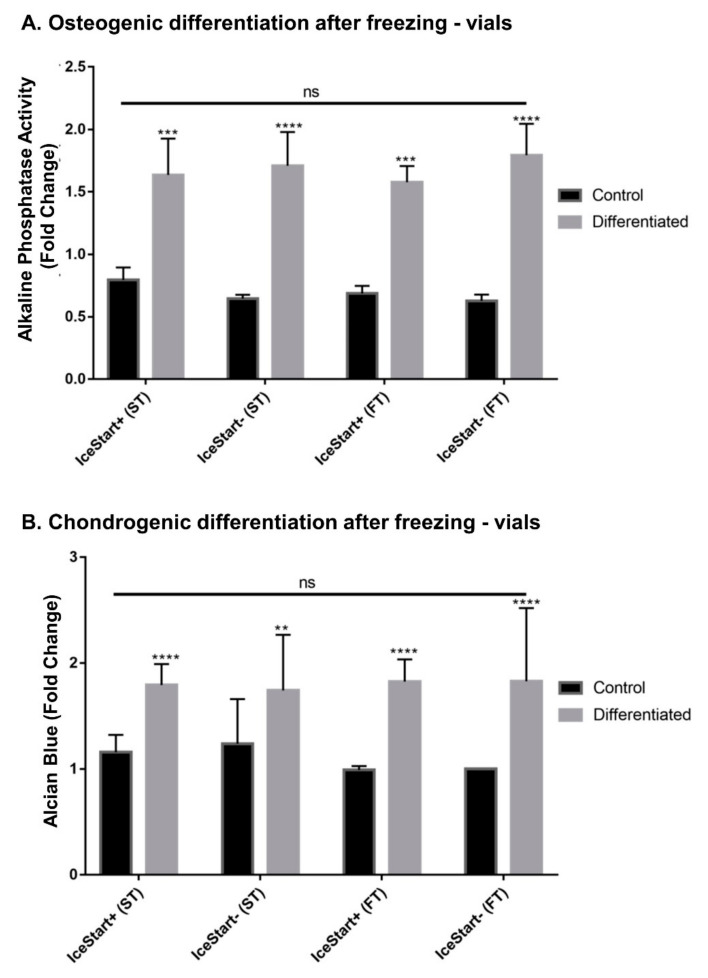
Quantitative analysis of osteogenic and chondrogenic differentiations. All differentiation conditions significantly greater than no differentiation controls with no significance between conditions. (** *p* ≤ 0.01, *** *p* ≤ 0.001, **** *p* ≤ 0.0001, bars represent ± SD *n* = 3 replicates + 3 donor repeats per condition.

Following differentiation protocols for vial conditions to ensure that the IND had no negative effects on the functionality of the MSCs, osteogenic, chondrogenic and adipogenic differentiation was observed (Figure 6 and Figure 7). The IceStart had no negative effect on the differentiation ability of the MSC.

## 3. Discussion

Cryopreservation is an important part of any cell therapy supply chain, for both commercial and research purposes. With the potential positive effects of especially allogeneic MSCs in many different diseases, effective long-term storage is critical. Current cryopreservation methods store cells in suspension in cryopreservation solutions below −135 °C [6]. The process of cooling and thawing, as well as the influence of the cryopreservation agents, can lead to damage and alteration of the desired function of the MSCs [7,8]. Cooling rate is one of the largest influencers in a successful cryopreservation process; freezing at a rate that is considered too slow causes dehydration of the cell with associated stresses and increases the osmotic imbalances as the solutes are excluded from the ice crystals potentially exposing cells to toxic concentrations, although the possibility of intracellular ice is reduced [6,22,25]. This can also lead to oxidative stress through reactive oxygen species (ROS) overwhelming the antioxidant mechanisms within cells, resulting in molecular damage to DNA, proteins and lipids [26]. Further, this can result in release of apoptogenic factors which can eventually lead to cell death. With freezing that is too rapid, osmolality is more balanced but intracellular ice is increased. Generally, a uniform cooling rate of −1.0 °C/min is accepted as effective for most cells, although exceptions exist [27]. Within the cooling process, variations in the temperature of ice formation, due to the stochastic nature of supercooling, can cause rapid fluctuations in the cooling rate from latent heat energy release; the lower the nucleation temperature, the greater the rate of change. The effect of an inappropriate basic cryopreservation process is normally observed within the first 36 h following thaw [10].

In the delivery of bespoke or commercialized therapies, a reduction of variations in the cryopreservation process (freezing and thawing) will reduce overall manufacturing costs and dosages by enabling a greater yield of functional cells at patient exposure. Additionally, the use of high-throughput methods (96+ well plates) in cell exposure testing (for example, in drug or CPA discovery) would benefit from off-the-shelf platforms with cells already attached and ready for culture. Exposure to rapid changes in temperature can stress cells and result in changes in material properties causing damage and detachment to cell populations [28].

Ice nucleation occurs from rough surfaces, storage solutions impurities (such as salts) and physical disturbances in the storage solution. By positively controlling ice formation temperature, cell recovery may be enhanced by reducing the variation in the rate of change of temperature during cooling and improve cell recovery and survival. Previous literature has reported spontaneous ice nucleation occurring at around −10 °C in cryovials up to 5.0 mL [29,30,31], in droplet freezing studies [32] and in plant sample studies [16]. This is corroborated by the data shown here in which the conditions without an IND in both 1.8 mL cryovials and in 96-well plates exhibited ice nucleation below −8.9 °C regardless of liquid volume. In the literature, a number of different methods are used to induce ice formation [11,12,13,15,33,34,35,36,37,38]. Many of these methods are unsuitable for use with advanced therapy medicinal products (ATMPs) as they are non-sterile, labour or cost intensive or would result in damage to the cells by other means.

Once methods of active induction of ice nucleation were employed in both cryovessels here, ice nucleation was induced at a significantly higher temperature resulting in more stable cooling rates. However, the exact nature of the cooling had different characteristics for 96-well plates and cryovials, despite previous reports of only a weak correlation between volume and nucleation properties [13]. Within cryovials, the addition of the IND resulted in a 10-fold decrease in variation. Contrastingly, the nucleation temperature range within the plates remained constant despite the IND. A similar result was reported when using the Snomax ice nucleating agent in which the uniformity of ice nucleation did not change [33]. Cambell and Brockbank (2014) also report that larger volumes enable more consistent cooling at slower rates [33].

Additionally, whilst individual wells in a 96-well plate act as isolated cryovessel in biological terms, they have demonstrated effects from a shared thermal environment which is exacerbated by the addition of the IceStart Array; well position had a significant effect on the ice nucleation temperature. This effect is of no surprise considering the increased mass surrounding each well, increasing the latent heat of the system [39]. Despite this, no significant well position effects were observed in cell recovery indicating a variable nucleation temperature is not necessarily as important as the temperature above which ice formation occurs.

Standard practice for cell recovery in cryovials is to encourage fast thawing utilizing a 37 °C water bath [23,24,35,40]. However, optimal heating rates within 96-well plates have yet to be defined. In addition to the IND, two thawing rates were investigated with both vials and plates in this study; fast thawing at 37 °C using the controlled rate freezer’s heating function in direct contact with the contact plate; and slow thawing in a cell culture incubator with the ambient temperature set to 37 °C. As supported by the initial human osteosarcoma cell line (SAOS-2) cells work using IceStart Arrays in 96-well plates [41], recovery was enhanced with the use of an IND. The slower thaw condition demonstrated the greatest recovery although the IND enabled the fast thaw condition to have a similar recovery to slow thaw IND- conditions. Analysis of viability on thaw and after one passage as well as the population doublings at passage 1 (Day 4) in vials also demonstrates an enhancement in recovery in 1.8 mL cryovials. Similarly to the 96-well plate condition, the IND was able to compensate for a less optimal thawing rate. Viability at thaw was greatest in the FT IND+ conditions, but FT IND- conditions and ST IND+ conditions demonstrated no significant differences, further suggesting that the use of IceStart in the cryopreservation process is capable of compensating for sub-optimal cryopreservation processes.

Campbell and Brockbank (2014) also investigated thawing rates in 96-well plate cryopreservation and 23 °C ambient temperature until −20 °C then rapid thawing at 37 °C [33]. The plates were then transferred to ice for washing before standard culture. Comparisons between the results of that study and the results presented here are difficult due to the relative presentation of the data, however the processes and tools presented in this study are less labour intensive. Campbell and Brockbank did report that recovery was greatest towards the edges of the plate, in contrast to this study in which recovery was higher towards the centre in IND+ conditions although as stated, not statistically significant.

Previously reported within the literature, MSC functionality, as measured through tri-lineage differentiation, has been negatively affected by the cryopreservation process although a recent review suggested that this trait is often conserved [8]. Utilizing a standard freezing and thawing process (without an IND and fast thawing in vials), differentiation was not shown to be negatively influenced in this study, however relatively low repeats may have disguised this. Comparison with the other conditions (IND+ and fast vs. slow thaw) also shows no difference, although in tandem with reducing variation in other measurements, it may be that any negative differentiation effects may occur less often as well.

## 4. Materials and Methods

### 4.1. Cell Culture

MSCs were isolated from human dental pulps (ethics approval was given by the ethics committee of the University of Leipzig, Medical Faculty—Study number 339-13-18112013, according to the Declaration of Helsinki of 1975. All human donors signed consent forms). Human dental pulp MSCs were expanded and incubated at 37 °C in normoxic conditions (air plus 5% CO2). MSCs were cultured in Dulbecco’s modified Eagle’s medium (DMEM, 1 g/L D-Glucose, Lonza, Slough, UK) with Glutamax, containing 10% fetal bovine serum (FBS, Gibco, Thermo Fisher Scientific, Dartford, UK) and experiments were undertaken between passages 6 to 8 (3 donors, age 18–25 years). Culture medium was exchanged every two days. MSCs were routinely passaged at 80% confluency by detaching using TrypLE Select (Gibco, Thermo Fisher Scientific, Dartford, UK) and centrifuging for 5 min (200× *g* at room temperature). Live cell numbers were determined using an Acridine Orange (Sigma-Aldrich, Gillingham, UK) (30 ug/mL)/DAPI (100 ug/mL) solution in dH2O, using the NucleoCounter NC-3000 (ChemoMetec, Allerød, Denmark). MSC were characterized by FACS for CD90, CD105 and C73 (positive markers) and CD14, CD20, CD34 and CD45 (negative markers) as well as tri-lineage differentiation before experimentation (Supplementary Figure S1) as defined by the ISCT [42].

### 4.2. Cryopreservation

#### 4.2.1. Freezing Protocol

**Figure 8:**
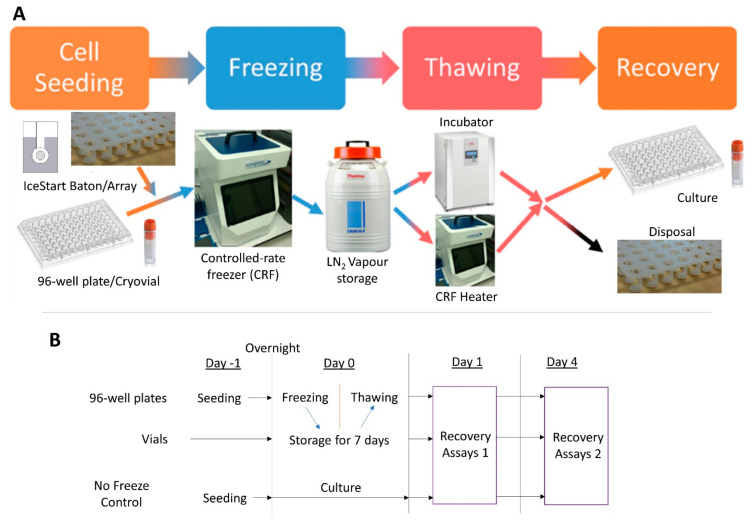
Experimental design schematic. (**A**) Cells are placed into vials or seeded into 96-well plates with cryostorage medium and ice nucleation devices (INDs). The cryovessels are then frozen in a controlled-rate freezer (CRF) before being transferred to liquid nitrogen (LN_2_) for extended storage. The cells were then thawed using either an incubator or the CRF’s heating capability before culturing and assessment of recovery. (**B**) Timings schematic for cryostorage culture assessment.

Freezing of 96-well plates and vials took place in a controlled-rate freezer (ViaFreeze, Asymptote, Cambridge, UK). The vessels were cooled at 1 °C/min until −75 °C and then transferred into the vapour phase of liquid nitrogen (less than −135 °C). The chamber of the freezing system (ViaFreeze) was pre-cooled to 4 °C before each experiment (Supplementary Figure S2). Cells were frozen in culture medium (described above) plus 10% dimethyl sulfoxide (DMSO, Sigma-Aldrich, Gillingham, UK) cryosolution. An experimental schematic is shown in Figure 8.

#### 4.2.2. Plates

MSCs were seeded at 4500 cells/well onto tissue culture plastic (polystyrene, Nunclon Delta, Thermo Fisher Scientific, Dartford, UK) and cultured overnight. Medium was replaced with 100 uL of cryosolution. A 96-well IceStart array (Asymptote, Cambridge, UK) (Supplementary Figure S2) was cut in half and placed into 48 wells of the 96-well plate and the plate transferred to freezing conditions.

#### 4.2.3. Vials

Once passaged, the MSCs were suspended in the cryosolution at 1.0 × 10^6^ / mL and then an IceStart baton was added to the test conditions (Supplementary Figure S2). The vials were then frozen as described above.

#### 4.2.4. Thawing Protocol

Frozen vessels were thawed either quickly (fast thaw, less than 8 min using the VIA Freeze) or slowly (slow thaw, circa. 30 min using an incubator). Following thaw, the IceStart array/baton was removed and, for 96-well plates, cryomedium replaced with culture medium (described above) following aspiration. One day post thaw, medium was again exchanged to remove any remaining traces of cryoprotectant. For vials, the MSC/cryosolution suspension was diluted in culture medium, centrifuged at 200× *g* at room temperature for 5 min, viable cells counted as above, resuspended in culture medium and seeded into 96-well plates at 4500 cells/cm^2^.

### 4.3. Cell Recovery (Metabolic Activity)

To assess the effect of position in 96-well plates with and without IceStart, an MTT reduction assay was performed on separate plates one and four days after thawing. After removal of culture medium, 100 μL MTT (0.5 mg/mL, Sigma-Aldrich, Gillingham, UK) in culture medium was added to each well and then left for 3 h at 37 °C in normoxic conditions. The supernatant was then removed leaving the reduced MTT formazan crystals, to which 100 μL DMSO was added to dissolve the crystals. After 30 min, the 96-well plate was read at 570 nm using a plate reader (FLUOstar Omega BMG LabTech, Aylesbury, UK) with reference at 630 nm.

To assess cell culture recovery, 100 μL of PrestoBlue Cell Viability Reagent (Thermo Fisher Scientific, Dartford, UK) at 10% *v/v* dilution in culture medium was added to each well at one- and four-days post thawing and incubated at 37 °C in normoxic conditions for 30 min. The plate was then read at 544 nm/590 nm (Excitation/Emission). For continued culture, the PrestoBlue solution was then replaced with culture medium.

### 4.4. Differentiation

Following thaw from vials, the cells were taken through one passage, and then seeded into relevant plates for quantitative and qualitative differentiation assays. A culture without application of respective differentiation media before staining provided a negative stain control (referred to as control in relevant figures).

#### 4.4.1. Osteogenic Differentiation

MSCs were seeded at 10,000 cells/cm^2^ into a 24-well plate. Medium was changed to osteo-inductive medium (low-glucose DMEM; 10% FBS; 10 nm dexamethasone, Sigma-Aldrich, Gillingham, UK; 50 µg/mL ascorbic acid 2-phosphate, Sigma-Aldrich, Gillingham, UK). Medium was changed every 2 days for a period of 14 days. For qualitative analysis of osteogenic differentiation, cells were fixed in 70% ethanol for 15 min and washed once with ddH2O. After washing, cells were stained with ALP buffer at pH 8.5 (0.2 M Tris, 1 mg/mL fast red, Sigma-Aldrich, Gillingham, UK and 50 µg/mL naphthol phosphate AS-BI, Sigma-Aldrich, Gillingham, UK) for 1 h and imaged under light microscopy.

#### 4.4.2. Adipogenic Differentiation

MSCs were seeded at 20,000 cells/cm^2^ into a 24-well plate. Cells were then cultured in Adipogenic medium (10% FBS; 10% insulin-transferrin-selenium supplement, Sigma-Aldrich, Gillingham, UK; 10^−8^ M dexamethasone, Sigma-Aldrich, Gillingham, UK; 0.5 mM isobutylmethylxanthin, Sigma-Aldrich; 100 µM indomethacin, Sigma-Aldrich, Gillingham, UK). The media was changed every 2 days and after 14 days cells were analysed by Oil Red O (Sigma-Aldrich, Gillingham, UK) staining and photos were taken.

#### 4.4.3. Chondrogenic Differentiation

MSCs were seeded at 50,000 cells/cm^2^ into a 12-well plate. Cells were then cultured in Chondrogenic media (10% FBS; 1% pen/strep 1% insulin-transferrin-selenium supplement (Sigma-Aldrich, Gillingham, UK), 10^−7^ M dexamethasone (Sigma-Aldrich, Gillingham, UK), 150 µM ascorbic-2-phosphate (Sigma-Aldrich, Gillingham, UK), 20 µM linoic acid (Sigma-Aldrich, Gillingham, UK) and 0.1 ng/mL TGF-β (Merck Millipore, Watford, UK). After 2 weeks, cells were stained with Alcian Blue (Sigma-Aldrich, Gillingham, UK) and photos were taken.

### 4.5. Temperature Profiles

Freezing and thawing profiles were measured using K-type thermocouples (RS components, Corby, UK). A TC-08 Thermocouple Data Logger and interface software (PicoLog 4, Pico Technology Cambridge, UK) were used to record the data.

### 4.6. Statistics

All experiments conducted with three donors for each assay (replicates stated for each figure). Statistical analysis was performed using GraphPad Prism 7 (GraphPad Software, USA). Significant differences were assessed using a two-way ANOVA with a Bonferroni post-hoc correction for data with significant F-ratio values. Statistical assumptions were satisfied unless stated otherwise.

## 5. Conclusions

This study demonstrates the use of a novel sterile, disposable IND for both cryovials and 96-well plate high-throughput platforms. In both vessels, the IND enhanced recovery and indicated an ability to compensate for sub-optimal cryopreservation processes. For cryovials, a faster thawing rate with an IND, resulted in greatest recovery and a reduction in undesirable variation between replicates. In 96-well plates, a slower thawing rate with an IND is more optimum. With no significant variation across the plate in cell recovery, this platform represents a viable method for HTS of CPAs and for delivery of an off-the shelf product for other studies, such as drug discovery.

## Supplementary Materials

Supplementary materials can be found at https://www.mdpi.com/1422-0067/21/22/8579/s1.
