# Supplementary material for: Cryopreservation of Mesenchymal Stem Cells Using Medical Grade Ice Nucleation Inducer
Source: Int J Mol Sci. 2020 Nov 13;21(22):8579. doi: 10.3390/ijms21228579 (PMC7696797; doi:10.3390/ijms21228579)

A. Controlled Rate Freezer Display

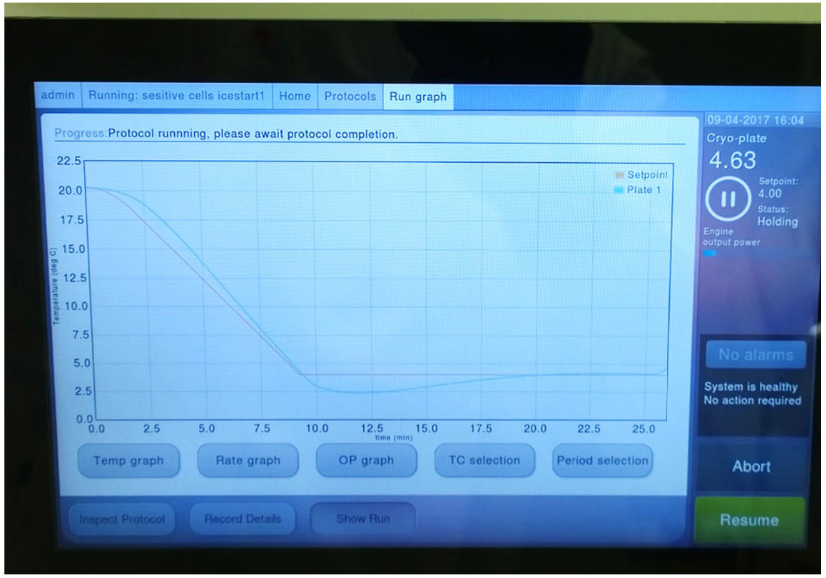

B. Measurement of Ice Nucleation Set-up

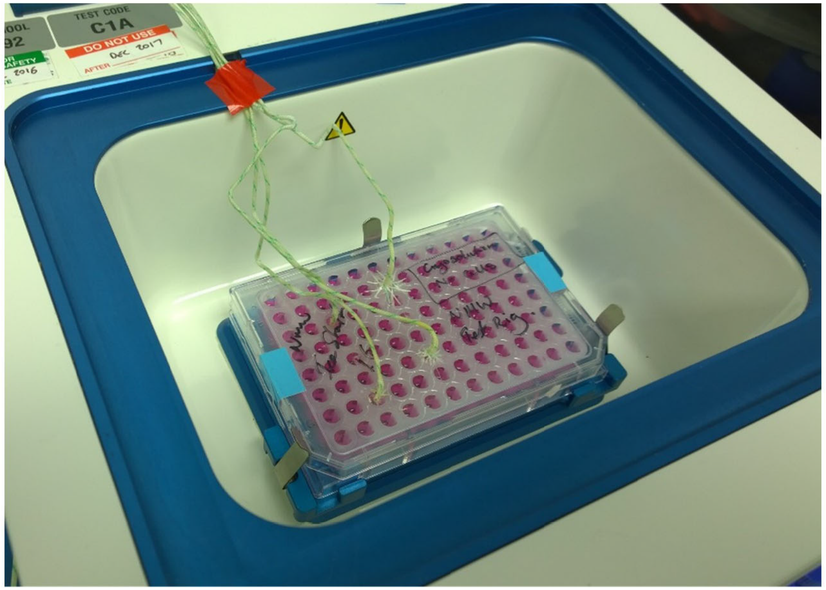

C. 96-well plate Ice Nucleation Array Comparison Set-up

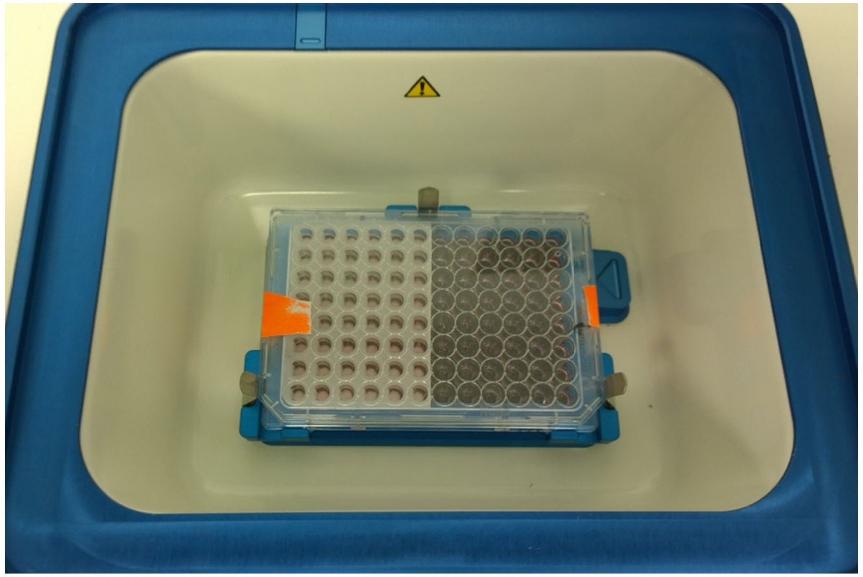

D. Schematics of Ice Nucleating Devices

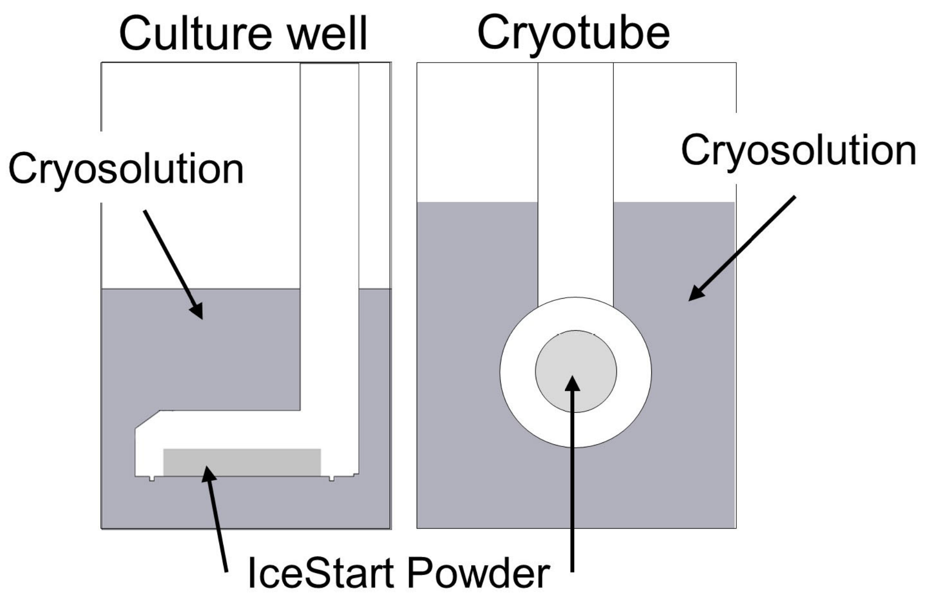

Supplement: Supplementary file 1 [file ijms-21-08579-s001.zip › ijms-943390-supplementary final/ijms-943390-Figure S2.pdf]
